# Real-Time Ultrasound-Guided Spinal Anaesthesia: A Prospective Observational Study of a New Approach

**DOI:** 10.1155/2013/525818

**Published:** 2013-01-10

**Authors:** P. H. Conroy, C. Luyet, C. J. McCartney, P. G. McHardy

**Affiliations:** ^1^Department of Anaesthesia, Adelaide and Meath National Children's Hospital, Tallaght, Dublin, Ireland; ^2^Universitätsklinik für Anästhesiologie und Schmerztherapie, Inselspital, Bern University Hospital, 3010 Bern, Switzerland; ^3^Department of Anesthesia, Sunnybrook Health Sciences Centre, Room M3-200, 2075 Bayview Avenue, Toronto, ON, Canada M4N 3M5

## Abstract

Identification of the subarachnoid space has traditionally been achieved by either a blind landmark-guided approach or using prepuncture ultrasound assistance. To assess the feasibility of performing spinal anaesthesia under real-time ultrasound guidance in routine clinical practice we conducted a single center prospective observational study among patients undergoing lower limb orthopaedic surgery. A spinal needle was inserted unassisted within the ultrasound transducer imaging plane using a paramedian approach (i.e., the operator held the transducer in one hand and the spinal needle in the other). The primary outcome measure was the success rate of CSF acquisition under real-time ultrasound guidance with CSF being located in 97 out of 100 consecutive patients within median three needle passes (IQR 1–6). CSF was not acquired in three patients. Subsequent attempts combining landmark palpation and pre-puncture ultrasound scanning resulted in successful spinal anaesthesia in two of these patients with the third patient requiring general anaesthesia. Median time from spinal needle insertion until intrathecal injection completion was 1.2 minutes (IQR 0.83–4.1) demonstrating the feasibility of this technique in routine clinical practice.

## 1. Introduction

Since the first description of spinal anaesthesia in humans by Bier in 1898 [1] the identification of the subarachnoid space has traditionally been achieved by an anatomical landmark guided approach. While surface anatomical landmarks are useful, they are nevertheless surrogate markers. They may be difficult to palpate in obese patients as well as those with edema. Landmark-based approaches do not take into account all anatomical variations or abnormalities, and frequently lead to incorrect identification of a given lumbar interspace [2]. Accurate identification of the subarachnoid space is paramount as multiple attempts at needle placement may cause patient discomfort, higher incidence of spinal hematoma [3], postdural puncture headache [4, 5], and trauma to neural structures [6–8]. Having alternative approaches may help improve success and mitigate the limitations of the current techniques. 

Neuraxial sonography allows the operator to preview spinal anatomy, identify midline, and determine the interspace for needle insertion. Even if the preliminary scout scan has already been shown to be helpful [9], a real-time ultrasound guided approach may further improve on the limitations of the ultrasound-assisted pre-puncture techniques. In particular this technique avoids the potential for error in the process of having to “remember” the degree of cephalad to caudad and lateral to medial angulation required after pre-puncture skin marking. To date we can find only one case report [10] and one report of a series of 10 patients [11] where the technique of real time ultrasound guided spinal anaesthesia has been employed. The aim of this single center prospective observational study was to investigate the feasibility of performing a systematic method for real-time ultrasound guided paramedian access to the subarachnoid space with the spinal needle inserted in the plane (in-plane) of the ultrasound beam by a single operator.

## 2. Methods

After ethical approval (Toronto Academic Health Sciences Network), registration at Clinicaltrials.gov (NCT01326988), and obtaining informed consent we recruited 100 patients scheduled to undergo spinal anaesthesia for elective lower limb surgery between May and September 2011. Other inclusion criteria were age 18 to 90 years, ASA I–III, and ability to provide written informed consent. Standard exclusion criteria for spinal anaesthesia included coagulopathy, INR ≥ 1.5, platelet count <75 × 10^9^/L, local infection at the site of injection, indeterminate neurologic disease, or allergy to local anaesthetics. Routine monitoring (ECG, heart rate, noninvasive arterial pressure, and arterial oxygen saturation) was established and intravenous sedation with midazolam (1-2 mg) was given. Any peripheral nerve block procedures appropriate to the patients' surgical procedure were performed prior to spinal anaesthesia (e.g., continuous femoral nerve block for knee replacement surgery). Spinal anaesthesia was administered either by a staff anaesthetist or one of 2 regional anaesthesia fellows, each with experience of more than 30 neuraxial ultrasound-assisted procedures. 

**Figure 1:**
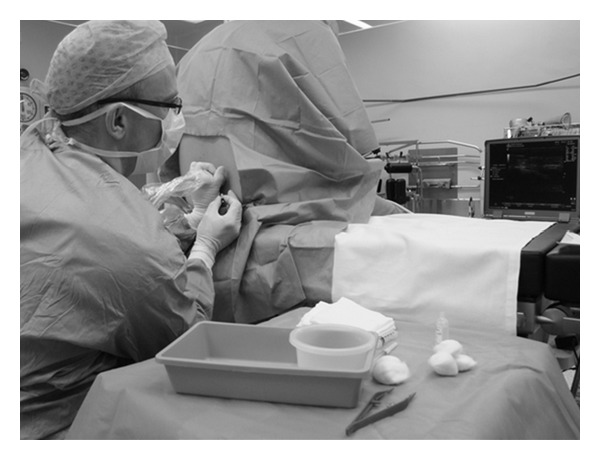
Typical positioning of the operator, ultrasound transducer and the patient during, real-time ultrasound-guided spinal anaesthesia.

 The block procedure was performed with the patient, at the operator's discretion, in the sitting or lateral position. Full aseptic technique with gloves, gown, mask, and sterile transducer cable sheath were employed. A standard curvilinear 2–5 MHz transducer attached to an ultrasound device (either a Sonosite M Turbo, Bothell, Washington, or a Phillips HD11Xe, Bothell, Washington) was applied to the patient's back, lateral to the midline (paramedian sagittal scan) in the preferred hand of the operator. The spinal needle was held in the opposite hand and the ultrasound screen positioned in the line of sight of the operator (see Figure 1). The US image was optimized by setting an appropriate scanning depth (6–10 cm), selecting a transducer frequency, and adjusting the gain to obtain the best possible image.

The sacrum was identified first and then the probe was moved cephalad in the paramedian axis with a 10–15 degree tilt toward the midline. The lumbar lamina were identified and a target space between L2-3 and L5-S1 was chosen. The probe was positioned with its midline point directly above the selected space. The transducer was then rotated 45 degrees towards midline, into an oblique, paramedian sagittal view (Figure 1). This maneuver affords a view of the spinous process of the upper vertebral body and the lamina of the lower vertebral body simultaneously. The angle between these two structures represents the paramedian window into the spinal canal. Selecting this approach reduces the risk of the facet joints obstructing the needle path as frequently occurs during a paramedian needle insertion within a transverse scanning plane. 

**Figure 2:**
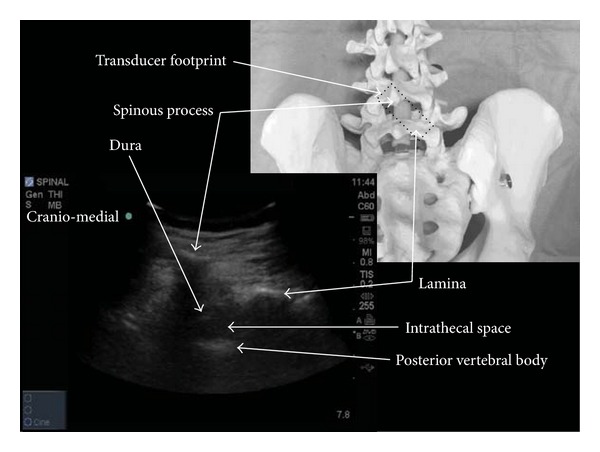
Spine model showing the paramedian oblique transducer orientation (rotated by 45 degrees) used during real-time scanning with a typical ultrasound image.

 With this view it is possible to visualize the lamina, the intervertebral space, and the posterior longitudinal ligament-vertebral body complex (seen as a white line deep in the intervertebral space). Unlike previous descriptions of real-time techniques, this maneuver provides an ergonomic advantage for simultaneous needle handling specifically because more room is created between the needle entry point and the bed surface. It also allows for a needle approach that more closely approximates the traditional paramedian technique as well as all the other advantages that a paramedian approach has been shown to afford when using neuraxial ultrasound [12]. As a final maneuver the probe is moved towards the patient's midline (craniomedial) in the same axis already established. The entry point of the needle would otherwise be too lateral when using a curvilinear probe. The final position of the probe and the image of the spine it provides can be seen in Figure 2.

**Figure 3:**
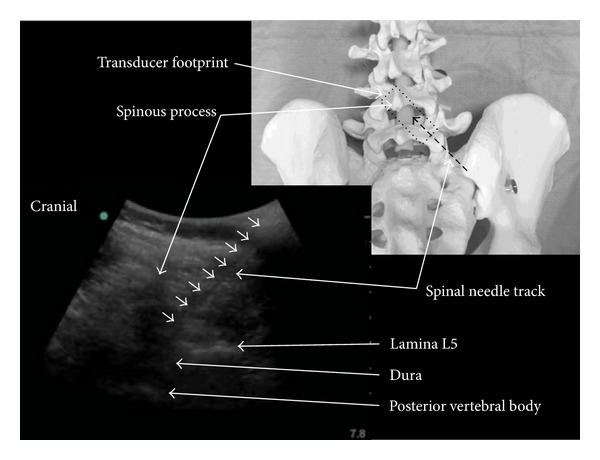
Ultrasound image showing the paramedian spinal needle insertion path compared with a schematic overlay of the transducer orientation on a spinal model.

Ultrasound gel was placed on the back directly under the transducer. Any ultrasound gel near the selected skin puncture site was carefully removed using sterile gauze prior to needle insertion. As a further precaution the point of needle entry through the skin was physically outside the footprint area of the transducer. Lidocaine 1%, (2-3 mL) was used for skin infiltration prior to the procedure. A standard spinal needle (90 mm long) was chosen at the discretion of the operator which provided the optimum balance of characteristics needed for ultrasound visibility and ability to steer in the tissues while at the same time considering the risk of postdural puncture headache. The needle was inserted in plane from the caudal end of the ultrasound transducer with its tip directed towards the interlaminar space. The angle of needle insertion, that is, the trajectory for needle insertion, was then optimized while the spinal needle was still in the erector spinae muscle (Figure 3). 

The needle was gradually advanced to the interlaminar space, under real-time in-plane US guidance, until the tip was judged to have broken through the ligamentum flavum/dura complex—frequently a distinct “give” could usually be felt at this point. Depending on the quality of the image and the size of the interlaminar space, the needle tip was occasionally lost from view if it entered the acoustic shadow formed by the spinous process. At this point the operator would feel the needle enter the ligamentum flavum and entry into the thecal space was typically achieved in less than one centimeter. With dosages varying according to the planned surgery plain 0.5% bupivacaine (5–15 mg) with fentanyl (5–15 *μ*g) was injected over 15–20 s and the patient was then returned to, or left in, the lateral position with the surgical side uppermost. The patient was determined to be ready for surgery at the discretion of the attending anesthetist. 

 Propofol (1-2 mg·kg^−1^·hr^−1^) was used for intraoperative sedation, and oxygen (4 L/min) administered via a facemask. The spinal anaesthetic was deemed to have failed if the procedure was converted to general anaesthesia within the first two hours after intrathecal injection. Postoperative care in the ward was entirely at the discretion of the orthopedic surgeons except for the analgesic management which was managed by the acute pain team.

The primary outcome measure was the success rate of CSF acquisition by real-time ultrasound guidance. Secondary outcome measures included the number of needle passes it took to access the subarachnoid space. Every ventral advancement of the needle was considered as a “needle pass” even if no further skin puncture was performed. Needle passes were limited to a maximum of 6 per skin puncture. Skin punctures were limited to a maximum of 3 per patient. The number of different spinal levels attempted, procedure time from first needle insertion to completion of the intrathecal injection and the number of separate skin punctures were recorded.

Scores were also recorded relating to the following aspects of the procedure

1. Preprocedure clinical scoring for potential predictors of difficulty in the event of a blind landmark based approach [13, 14].
2. Summary difficulty score according to the anesthetist's subjective opinion: Easy/Normal = 0, Difficult = 1.
3. Bony landmark palpability: 0 = easily palpable; 1 = poorly palpable; 2 = impalpable.
4. Presence of spinal curvature abnormality: None = 0; Yes = 1.

In the event that the permitted number of needling attempts was reached without acquiring CSF then the real-time US technique was defined as a failure. In such cases the spinal block was performed using either a traditional landmark based approach and/or pre-puncture ultrasound assistance at the discretion of the attending anaesthetist. Complications were recorded including those directly related to the technique (e.g., vascular puncture), inadequacies of the block that required rescue spinal injection of local anaesthetic, or conversion to general anaesthesia within 2 hours of the initial spinal anaesthetic injection. All study data were recorded by the operator after performing the procedure and patients were followed up by telephone at 6 weeks postoperatively. 

### 2.1. Statistics

A convenience sample of 100 consecutive patients was planned in order to represent the typical patient population presenting for elective spinal anaesthesia. Descriptive statistics were calculated for all variables of interest. Normally distributed data are presented as mean and standard deviation—non-normally distributed data are presented as median (interquartile range [range]). Normality was tested using the Shapiro-Wilk test. Correlation between Preprocedure clinical scoring for predictors of difficulty in the event of a blind landmark based approach and measurements made during the puncture such as total number of needle advancements, total number of skin punctures, and the time to completion of the intrathecal injection were calculated using Spearmans rank correlation coefficient for non-normally distributed data or the Pearson Product Moment correlation coefficient for normally distributed data. All calculations were carried out using Sigma Plot 12 for Windows (Systat Software Inc. GmbH, Germany). 

## 3. Results

100 patients were completed and patient characteristics are detailed in Table 1. Using real-time ultrasound guidance CSF was acquired in 97% of cases within the predefined limits set in the study protocol. There was failure to acquire CSF by real-time guidance in 3 patients. Subsequent attempts using a combination of the traditional landmark palpation method and pre-puncture ultrasound scanning resulted in successful spinal anaesthesia in 2 of these patients. The third patient received elective general anaesthesia following multiple failed attempts to acquire CSF using a combination of all the approaches described above by 2 different operators.

5% of patients who received intrathecal local anaesthetic injection were subsequently converted to general anaesthesia intraoperatively—2 patients had poor CSF flow during injection, 1 had no block despite 2 easy injections of LA into free-flowing CSF, 1 patient became very restless while sedated 50 minutes into a total knee arthroplasty, and 1 patient had inadequate block height for surgery with insufficient time to perform a repeat block.

The median number of needle passes required to acquire a satisfactory flow of CSF for spinal anaesthetic injection was 3 (IQR 1–6 [range 1–18]). The median number of skin punctures was 1 (IQR 1-2 [range 1–3]). 65% patients required only one skin puncture for CSF acquisition with 12% patients needing the maximum allowed three skin punctures to successfully complete the injection. The number of median spinal levels punctured was 1 [IQR 1-2]). 30% patients required only a single needle pass with CSF being obtained in 80% cases within 6 needle passes. An average of 1.46 spinal interspaces was punctured. 

 The median time required for sterile preparation of the ultrasound equipment and initial pre-puncture scanning was 3.9 mins (IQR 2.7–5.2 [range 1.4–15.3]). The median time required from spinal needle insertion until completion of the intrathecal injection was 1.2 mins (IQR 0.8–4.1 [range 0.2–15]). Total procedure time (excluding patient positioning and local anaesthetic preparation) was 8 minutes (IQR 4.4–10.2 [range 2.9–19.7]). Patients were in the seated position in 95% of cases (lateral position in 5 cases)—one of the 3 patients where real-time US guidance failed to locate CSF was in the lateral position. CSF was acquired using real-time needle guidance through a 22 G Quincke spinal needle in 86% of cases and a 25 G Whitacre needle in the remainder (all needles used were 90 mm in length). All patients were followed up by telephone interview at 6 weeks post procedure for procedure-related complications with a telephone response rate of 67%. An orthostatic post dural puncture headache was reported by one patient on whom a 22 G Quincke needle was used.

Data relating to the preintervention predicted difficulty of performing spinal anaesthesia are presented in Table 2. There was no significant correlation between the total number of skin punctures, time to acquire CSF, or the total number of needle advancements and either the patients BMI, the anaesthetist's subjective Preprocedure prediction of difficulty or the palpability of bony landmarks. There was, however, a significant correlation observed between the presence of a spinal curve abnormality and both the number of skin punctures required (correlation coefficient 0.251; *P* = 0.0124) as well as the total number of needle advancements required (correlation coefficient 0.307, *P* = 0.002). Overall 63% of patients recruited were predicted to be difficult according to the previously validated pre-procedural difficulty scoring system by Atallah et al. [13]. 

## 4. Discussion

This study demonstrates that real-time ultrasound guided spinal anaesthesia is a clinically feasible procedure among this unselected population of patients undergoing lower limb orthopaedic surgery. This a patient group among whom the greatest outcome benefits are to be gained from neuraxial block [15]. A success rate of 97% in CSF acquisition using real-time US guidance appears to be broadly similar to that achieved in other studies of the adult orthopaedic patient population using traditional landmark [16] or ultrasound guided pre-puncture [9] approaches for spinal anaesthesia. Administration of spinal anaesthesia under real time ultrasound guidance failed in 3 patients within the predefined limits specified in the real-time study protocol. 2 of these patients eventually had successful spinal anesthesia using a combination of traditional landmark palpation and ultrasound pre-puncture scanning outside of the real-time study protocol (i.e., according to routine clinical practice in our institution with no predefined limitations on the number of needle insertions/skin puncture attempts). As this was a non-comparative trial a definitive conclusion cannot be drawn from this data as to the relative merits of the real-time guidance method versus landmark only or ultrasound pre-puncture techniques. 

 Acquisition of CSF in and of itself does not necessarily reflect the number of needle passes or skin punctures required to achieve this endpoint and thus may not reflect the actual procedural difficulty encountered—assessment of the usefulness of this technique according to these clinically relevant outcomes requires a prospective randomised trial comparing these 3 different methods for the administration of spinal anaesthesia. 

Use of real-time ultrasound guidance for combined spinal-epidural insertion as part of a randomised controlled trial in a younger obstetrics population has previously found that real-time guidance significantly reduces the number of needle passes required when compared to a traditional landmark based approach [17]. Our success rate for correct identification of the subarachnoid space on the first skin puncture (65%) is similar with others previously reported (64% [14], 61.5% [18], and 61.9–68.3% [19]). Minimising the number of skin punctures is important due to the risks associated with multiple attempts at needle placement as described earlier (i.e., a higher incidence of spinal hematoma, postdural puncture headache, and trauma to neural structures) [3–8].

Patients were in the sitting position in 95% cases (5% were in the lateral position) which is typical of our pre-existing institutional practice. Performing real-time US guided spinal anaesthesia in the prone position as reported by Lee et al. [11] can be difficult for patients to tolerate and introduces other practical issues. In this study, choice of patient positioning was left to individual operator discretion who found both the lateral and sitting positions to be ergonomically feasible. 

 Total procedure time was 8 ± 4.68 minutes (average ± SD). Typical procedure times reported for a landmark based approach range from 4.4 ± 3.2  mins [19] to 4.8 ± 4.4 mins [16]. Real-time US guided spinal anaesthesia takes longer because extra time is required to identify a satisfactory acoustic window coupled with the need to maintain needle-probe alignment. The time spent for sterile preparation of the probe and initial “scout” scanning prior to insertion of the needle (average 4.4 min ± 2.6 min) appears longer than that reported for ultrasound assisted neuraxial techniques by Carney and Hunt (2.7 min (±0.6 min)) [20]. However the mean time taken for real-time scanning of the spinal needle approach until completion of the intrathecal injection was an average of 3 min (±3.5)—in effect this was the duration of combined real-time scanning and “needling”. 

### 4.1. Failure Rate

Failure of spinal anaesthesia has been defined as the need to convert to general anaesthesia or to repeat the intrathecal injection following the initial block—this occurred in 5% of patients in this study. There are widely varying reports of the failure rate of spinal anaesthesia in the literature ranging from <1% to 17% [21]. Contemporary reports of spinal anaesthesia failure rate continue to be in the range of 11.6% [16].

Our technique had a 97% success rate of CSF acquisition in this study. The only other real-time ultrasound guided neuraxial procedures in which this outcome was reported were in two studies of combined spinal-epidural anaesthesia which had failure rates of 22% (4/18 patients [22]) and 14% (2/14 patients [23]), respectively. However given the different nature of the techniques and different reasons for failure (as detailed in a recent review by Cook [24]) direct comparison is not possible. 

### 4.2. Difficult Spinal Insertion

The ease of palpation of spinal bony landmarks (classified as easy, poorly, or totally impalpable spinous processes) has been shown to be the most significant independent predictor of difficulty with spinal anaesthesia administration in two previous studies [13, 14].

Atallah et al. have previously developed a difficulty scoring system for predicting difficult spinal anaesthesia where patients are given a score ranging from 0 to 4 for each of the variables age, BMI, surface landmark palpability, spinal bony deformity, and radiological characteristics [13]. A score ≥4 is predictive of difficulty even if the radiological score is omitted. By this yardstick 63% of our patients were predicted to be difficult. However on the other hand the average BMI of this sample of patients was not very different to that of average North American adults in the 40–79 year old age range. The mean BMI of adults in this age range in Canada for the period 2007–2009 was 27.7 kg/m^2^ while that of Americans was 29.1 kg/m^2^ [25]. In other words our patients were not always “difficult” and further data are needed for difficult patients in which traditional landmark or even pre-puncture scanning may prove to be inferior to real time ultrasound guidance.

Conversely using our guidance technique only 7% of spinal insertions in this study were found to be “difficult” according to separate difficulty criteria proposed by Weed et al. (i.e., ≥10 needle passes associated with spinal needling time of >400 seconds or cases where CSF failed to be obtained) [26]. In contrast Weed et al. found 28% of their patients to be difficult by these criteria when using a landmark based approach. This apparent reduction in predicted difficulty when using our technique could be due to the improved visualisation and guidance of the needle path offered by real-time scanning as opposed to a pre-scan or landmark technique.

### 4.3. Spinal Needle Size

An audit of spinal anesthesia practice was conducted 1 year prior to this study at our institution. Data collected relating to the size of spinal needles used at that stage indicates 25 G Whitaker needles were used in 81% of our patients (a 22 G Quincke was used in the remainder). This is markedly different from the pattern in our study where we used 22 G needles in 96% patients. This change was motivated by the fact that 22 G needles are stiffer and more echogenic than finer 25 G needles thereby making them easier to visualise and to steer within the ultrasound beam towards the target. This consideration however can be obviated by using a longer introducer needle with corner-stone ultrasound reflectors or indeed by using a 22 G needle as an introducer and then stopping short of the dura before inserting a 25 G needle through the lumen as suggested by Medd et al. [27]. However despite our predominant use of 22 G needles only one patient reported postdural puncture headache during follow-up giving an estimated incidence of 1%. 

### 4.4. Study Limitations

Our study is a single centre descriptive observational study of real-time US guided spinal anaesthesia and not a comparative randomised controlled trial. We cannot, therefore, make any direct comparisons between this approach versus a pre-puncture ultrasound or landmark only technique. To this end we are currently planning a randomised controlled trial comparing these techniques directly paying particular attention to that subset of patients with a high Preprocedure difficulty score according to Atallah et al. 

It was not practicable to blind the study and the individual operators self-reported the study outcomes directly following performance of the procedure. This introduces the possibility of observer bias [26]. 

## 5. Conclusion

In summary we have demonstrated that real-time ultrasound-guided spinal anaesthesia is a clinically feasible procedure. Performance of the technique assessed across a broad range of measures appears similar to the traditional landmark based technique as well as pre-puncture ultrasound assisted approaches.

## Figures and Tables

**Table 1:** Patient characteristics.

		Surgery performed (*n*)	
Total Patients (*n*)	100		
Gender (*n*)	M: 37, F: 63	Total knee arthroplasty	61
Age (yrs)	66 ± 11	Total hip arthroplasty (THA)	24
	Range 39–86		
Height (m)	1.7 ± 0.1	Revision THA	3
	Range 1.7–1.9		
Weight (kg)	83.5 ± 18.1	Other knee surgery	4
	Range 45–146		
BMI (kg·m^−2^)	30.4 ± 5.9	Other lower limb surgery	9
	Range 17.6–52.1		

Values expressed as number *n* (= % patients) or mean ± SD.

**Table 2:** Predicted difficulty of spinal anaesthesia.

Anaesthetists subjective prediction of Difficulty	Easy	64
	Difficult	36

Palpability of bony landmarks	Easily palpable	53
	Poorly palpable	35
	Impalpable	12

Predicted spinal anaesthesia difficulty scoring (after Atallah)	Easy (grade 0–3)	63
	Easy (grade ≥4)	37

Data are presented as *n* (= %). Atallah's spinal anaesthesia difficulty score comprises nine difficulty grades. Grade 4 is the difficulty grade at or above which difficulty is expected. Grades are calculated based on the sum of specific scores assigned for BMI, age, bony landmark palpability, radiological characteristics, and spinal deformity [13].
